# Supplementary figures and images for: Leveraging molecular-QTL co-association to predict novel disease-associated genetic loci using a graph convolutional neural network
Source: PLoS One. 2025 Jun 10;20(6):e0324183. doi: 10.1371/journal.pone.0324183 (PMC12151363; doi:10.1371/journal.pone.0324183)

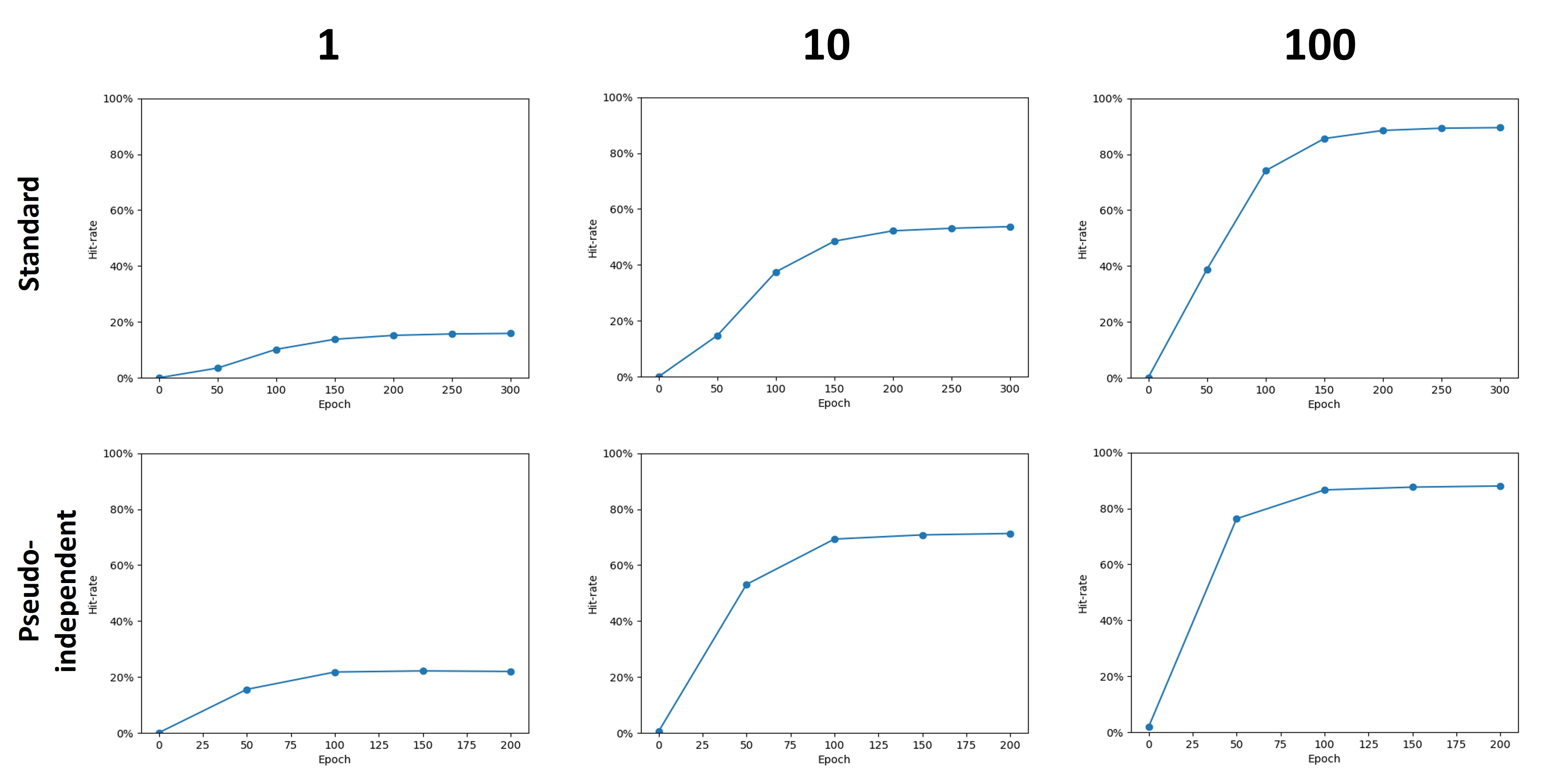

Supplement: S1 Fig — Line graphs showing the improvement in Model 1 hit-rate over time when 1 (left), 10 (middle) or 100 (right) recommendations are made. Model performance is provided for both the standard (top) and pseudo-independent (bottom) embeddings. The hit-rate was evaluated every 50 epochs. The initial hit-rate (epoch 0) is based on initialised model weights (JPG) [file pone.0324183.s001.jpg]

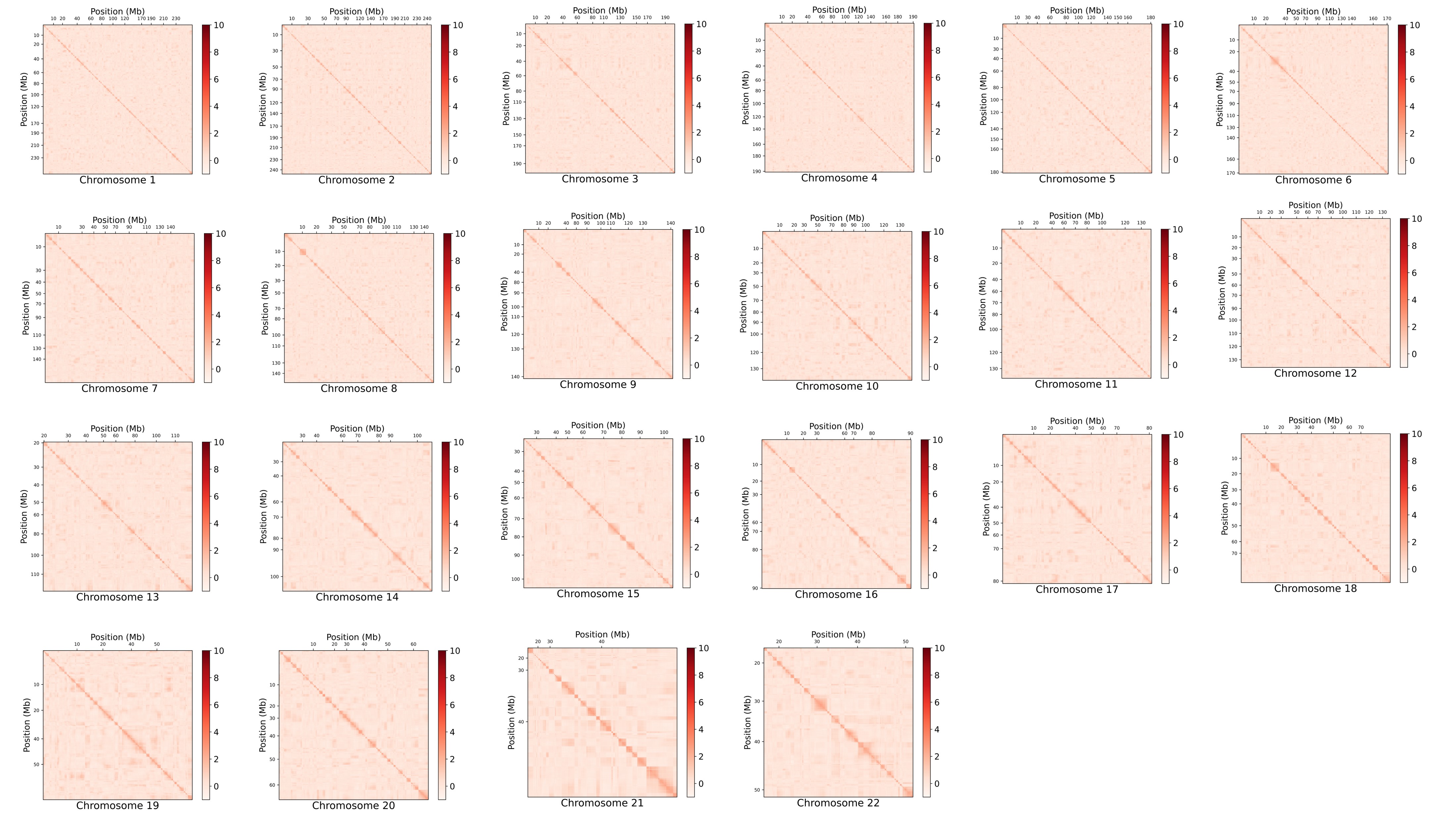

Supplement: S2 Fig — Heatmap of the similarity matrix representing functional interaction across SNPs, for all 22 chromosomes. Genome position is on both axes. Higher values (darker red) indicate greater similarity between two loci, similarity here being given by the dot product of their respective embeddings. The diagonal represents self-similarity. High similarity scores closer to the diagonal represent the local (cis) region; whilst those further away from the diagonal represent trans-region(s) co-associating together. The similarity matrix is based on the pseudo-independent embedding. (JPG) [file pone.0324183.s002.jpg]
